# An investigation of Reablement or restorative homecare interventions  and outcome effects: A systematic review of randomised control trials

**DOI:** 10.1111/hsc.14108

**Published:** 2022-12-02

**Authors:** Cate Bennett, Francis Allen, Sevim Hodge, Phillipa Logan

**Affiliations:** ^1^ School of Medicine: Ageing and Rehabilitation Nottingham University Nottingham UK

**Keywords:** home care, occupational therapy, RCT, reablement, restorative, systematic review

## Abstract

The effect of Reablement, a multi‐faceted intervention is unclear, specifically, which interventions improve outcomes. This Systematic Review evaluates randomised controlled trials (RCTs) describing Reablement investigating the population, interventions, who delivered them, the effect and sustainability of outcomes. Database search from inception to August 2021 included AMED, ASSIA, BNI, CINHALL, EMBASE, HMIC, MEDLINE, PUBMED, PsycINFO, Google Scholar, Web of Science, Clinicaltrials.gov. Two researchers undertook data collection and quality assessment, following the PRISMA (2020) statement. They measured effect by changed primary or secondary outcomes: no ongoing service, functional ability, quality of life and mobility. The reviewers reported the analysis narratively, due to heterogeneity of outcome measures, strengthened by the SWiM reporting guideline. The search criteria resulted in eight international studies, five studies had a risk of bias limitations in either design or method. Ongoing service requirement decreased in five studies, with improved effect at 3 months shown in studies with occupational therapist involvement. Functional ability increased statistically in four studies at 3 months. Increase in quality of life was statistically significant in three studies, at 6 and 7 months. None of the studies reported a statistically significant improvement in functional mobility. Reablement is effective in the context of Health and Social Care. The outcomes were sustained at 3 months, with less sustainability at 6 months. There was no statistical result for the professional role regarding assessment, delivery and evaluation of interventions, and further research is justified.

What is known about this topic?

1. Reablement is cost‐effective.
2. Reablement can improve people's independence outcomes.
3. Reablement is a multi‐faceted complex intervention.

What does this paper add?

1. Reablement interventions are heterogenic, in terms of the timescale of delivery, and dose, and this influences outcomes.
2. Interventions delivered during Reablement can reduce the need for ongoing home care and improve activities of daily living and quality of life at three to four months, with sustainability beyond 6 months.
3. Professional role for assessment, delivery or evaluation of Reablement progress did not have a statistical consequence on outcome effect, and therefore cannot be generalised, this justifies further research.

## 1 INTRODUCTION

Reablement is restorative home care supporting individuals physically, socially and psychologically to regain the health, skills and independence required for daily living (Clotworthy et al., 2021). As defined by an International Delphi Study,

"Reablement is a person‐centred, holistic approach that aims to enhance an individual's physical, and or other functioning, to increase or maintain their independence in meaningful activities of daily living at their place of residence, and to reduce their need for long‐term services" (Metzelthin et al., 2020 p11).

Reablement, valued for its potential to decrease demand for home care (Cochrane et al., 2016), facilitate hospital discharge, and its cost‐effectiveness (Francis et al., 2011; Kjerstad & Tuntland, 2016; Tuntland et al., 2017). People who benefit most have mild or moderate frailties or live alone (Lewin et al., 2013). Furthermore, Reablement has no impact on the burden of informal caregivers compared to standard care (Senior et al., 2014).

The ethos of Reablement is that the carer works ‘with’ the person to achieve goals, rather than doing the activity ‘for’ the person (Metzelthin et al., 2020; Tew et al., 2014). The ideal Reablement harnesses strengths and has a highly functional and social connectivity focus (Doh et al., 2020), with meaningful and achievable goals, focusing on what matters to the person (Social Care Institute of Excellence (SCIE, 2020).

Reablement involves multi‐faceted interventions (Metzelthin et al., 2020; Whitehead et al., 2018), and can be time‐limited (Clotworthy 2021). Past systematic reviews indicated a need to evidence the effectiveness of Reablement interventions (Boniface et al., 2013; Legg et al., 2015). Reablement interventions are usually, but not exclusively, provided by Occupational Therapists to enable self‐care activities: Activity analysis, motivational coaching, practising skills, risk enablement, compensatory techniques, assistive technology, equipment and adaptations (SCIE, 2020; Whitehead et al., 2018; Zingmark et al., 2020). Activity analysis is a unique Occupational Therapist skill (Thomas, 2012). Tuntland et al. (2020) found that mobility was a key priority for particpants goals regardless of their health condition.

The academic literature is unclear on the specific professional roles involved in Reablement (Metzelthin et al., 2020; Pettersson & Iwarsson, 2017). The most successful Reablement according to SCIE (2020) has occupational therapy input. Royal College of Occupational Therapists (RCOT, 2019) argue that Occupational Therapists are best placed to deliver specialist and complex Reablement interventions and to supervise or train others due to the scope of their professional training.

Reablement service delivery models vary depending on organisational constructs. Beresford, Mann, et al (2019) conducted a UK survey reporting 53% of Reablement services were delivered by the Local Authority, and 17% included Occupational Therapists. Internationally, Reablement has element of restorative home care (Metzelthin et al., 2020). The literature evidences Reablement is multi‐disciplinary (Tuntland et al., 2015), Nurse‐Led (Metzelthin et al., 2017; Rooijackers et al., 2021), and non‐specific care management (King et al., 2012; Parsons et al., 2013). Where teams have multi‐speciality roles, Zingmark et al. (2020) found the focus and content of interventions indicated by professional role contributions were complementary. Where studies are non specific about professional qualification or training background, it raises a problem as it is difficult to establish the optimum professional role or skill mix to deliver Reablement effectively.

This review aims to identify, critically appraise, analyse and evaluate the results of peer‐reviewed Randomised Control Trials (RCTs) examining the effectiveness of interventions used in Reablement. to establish a clear link between evidence‐based practice and the intervention,

1. To describe the Reablement interventions, who delivers them, how and when,
2. To critically evaluating the effects on an individual's progress, during and/or after a period of Reablement.
3. To investigate whether there is any lasting or long‐term outcome of any Reablement interventions.

## 2 MATERIALS AND METHODS

### 2.1 Search strategy

The research question was based on the PICO framework, population, intervention, control and outcomes (Thomas et al, 2022). The reviewers used the Preferred Reporting Items For Systematic Reviews and Meta‐analyses (PRISMA) (Page et al. 2021) to report the search, documented in the PROSPERO registered Protocol (CRD42021237209). The search was for peer reviewed, published RCT on adults receiving Reablement interventions, as opposed to standard home care, where there was a between group difference on outcomes measured. The date range was from database inception to 31 August 2021. There was no restriction placed on language, country and historical date. There were no studies found in languages other than English.

The search terms:

1. ‘Occupational thera*’ and (‘reablement’ or ‘rehabilitation’ or ‘restorative’) and (‘RCT’ or ‘randomised control trial’)
2. ‘Occupational thera*’ and (‘reablement’ or ‘rehabilitation’ or ‘restorative’) and (‘home care’ or ‘home care provider’) and (‘RCT’ or ‘Randomised control trial’)
3. (‘reablement’ or ‘restorative’) and (‘RCT’ or ‘randomised control trial’)
  The review included consenting adults over 18 years, receiving Reablement home care or receiving an occupational therapy service designed to align with standard home care. The reviewers excluded studies if particpants had no home care service, were residential or nursing home dwelling, palliative diagnosis, diagnosis of dementia or Mini Mental score of less than 18.
  The database search included AMED, ASSIA, BNI, CINAHLL, EMBASE, HMIC, MEDLINE, PsycINFO, PubMed, Google Scholar, Web of Science and Clinicaltrials.gov. A hand search of references, grey literature and a Zetoc email alert ensured thoroughness.
  Two reviewers independently undertook the search, critically appraising the studies for eligibility using a recognised tool (Critical Apporaisal Skills Programe (CASP, 2021), and disagreements were resolved through reflection, careful re‐examining of the data and discussion. The full search results with inclusion and exclusion reasoning is available in Appendix S1. All completed data collection forms and other study information are available through correspondence with the author.
  The synthesis method involved a three‐stage process. Firstly, the interventions and comparisons were established in the protocol.
  Secondly, the reviewers completed the data extraction process establishing the characteristics of each study,  tabulating each outcome (Appendices S2 and S3) enabling comparison. The ‘intention to treat’ outcome effect was used to evidence assignment to the intervention, missing data reported in the risk of bias analysis (Higgins et al., 2020).
  The reviewers used the revised Rob2 risk of bias framework tool (Cochrane, 2021) to establish the internal validity of the studies.The tool has signalling questions with detailed explanations and an embedded answer algorithm to determine the level of concern about issues that are likely to affect the rability to draw reliable conclusions from the study. The risk domains are the randomisation process; deviations from intended interventions; missing outcome data; outcome measurement; and selection of the reported results. In addition, the reviewers used the supplementary questions for cluster RCT trials (Eldridge et al., 2020).

The Rob2 criterion for overall risk of bias is based on a combination of the embedded algorithm and assessor judgement, the assessor's decision has the final influence on risk weighting. Higgins et al. (2020) define the criteria as follows:

Low risk of bias: all domains were judged low risk.

Some concerns: at least one domain had a concern, but not a high risk of bias in any domain.

High risk of bias: either the high risk of bias in one domain or some concerns in multiple domains.

Finally, stage 3 pulled the strands of the analyisis together to deterime the quality. Two reviewers discussed and determined each risk decision, including the direction and strength, giving certainty of bias for each study, strengthening the link between study design and resulting intervention effects (Sterne et al., 2019). The cumulative evidence synthesis was coherently summarised to determine that the true effect lies within a particular range or side of a threshold, using the definitions established by the Grade, Recommendations, Assessment, Development and Evaluation (GRADE) working group (Hultcrantz et al., 2017).

The reviewers strengthened the reporting of the synthesis by using SWiM (Campbell et al., 2020), a framework designed to improve narrative reporting of the analysis process, supported by visual data to describe the range, distribution and effects (Robertson‐Malt, 2014).

### 2.2 Declaration of sources of funding

The primary author is funded for a PhD study by the National Institute for Health Research Applied Research Collaboration East Midlands, United Kingdom.

## 3 RESULTS

**FIGURE 1:**
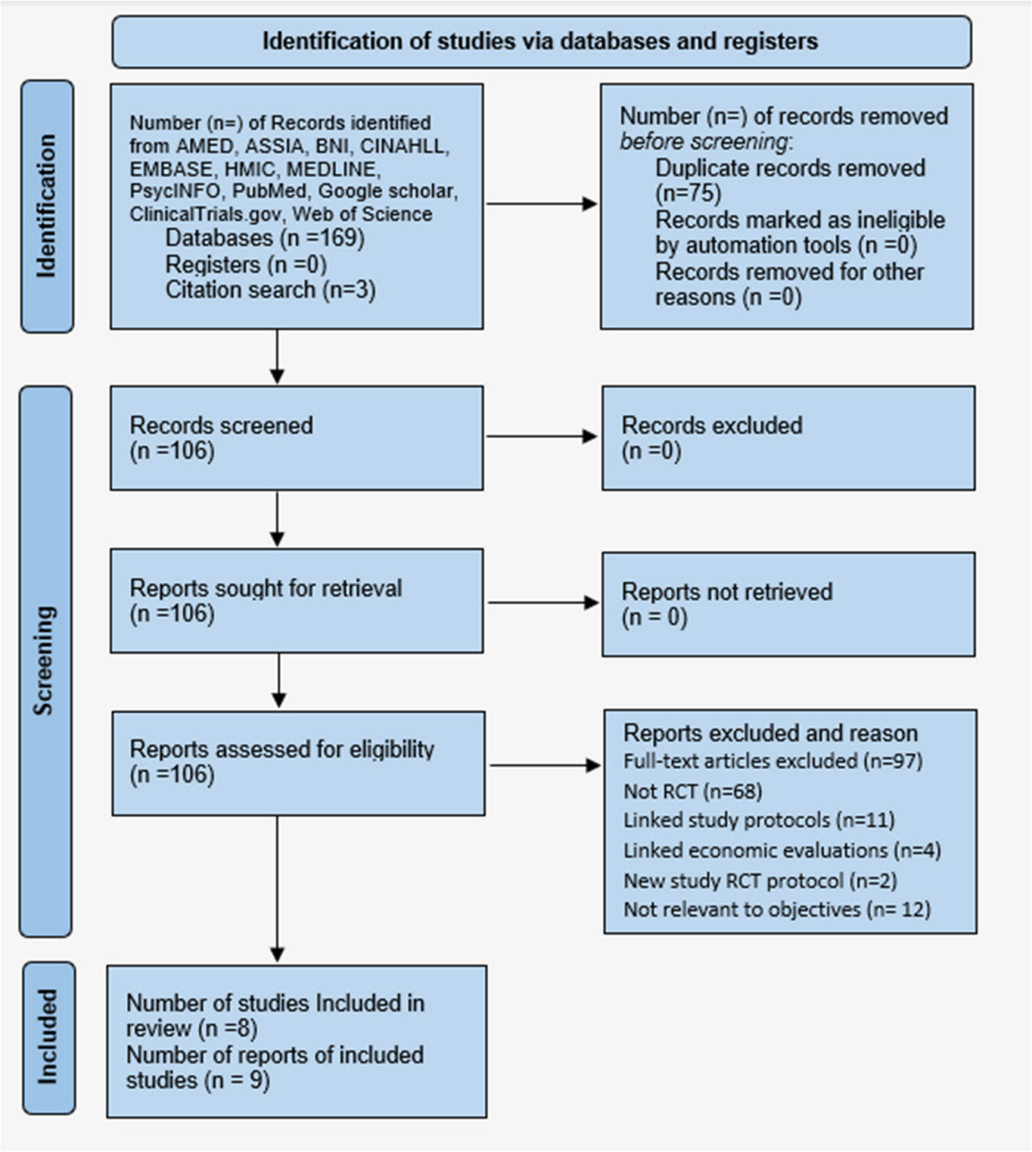
Literature search strategy. Prisma (2020) Systematic review search from databases and registers (Page et al., 2021)

The reviewers found nine journal articles, reporting eight studies from the literature search strategy, presented using the PRISMA (2020) flow diagram for systematic reviews, Figure 1 (Page et al., 2021).

Two published journal articles (Parsons et al., 2012; Parsons et al., 2013), reported the same registered RCT, both are included in the analysis to capture the risks associated with the secondary outcome measure documented in Parsons et al. (2013). Reasons for other studies’ exclusion are available in Appendix S1.

### 3.1 Population

The mean age of 1777 participants included in the studies reviewed was 80.35 years. All had difficulty completing activities of daily living at home, requiring a home care service.

Age, over 65 years, was an inclusion criterion in five studies. In addition, Parsons et al. (2012, 2013) included over 55 years old from Māori or Pacific Islander ethnicity, and Tuntland et al. (2015) and Whitehead et al. (2016) included adults over 18 years old. All studies included more females than males (Table 1). People who lived alone, reported in six studies (Table 1), showed a higher incidence in the intervention group for all studies, except Burton et al. (2013).

**TABLE 1:** Population characteristics at baseline

Study	Age		Gender				Lives alone	
	Intervention n (SD)	Control *n* (SD)	Intervention		Control		Intervention *n* (%)	Control *n* (%)
			Female *n* (%)	Male *n* (%)	Female *n* (%)	Male *n* (%)		
Sheffield et al. (2013)	81.05 (±9.06)	82.43 (±10.01)	32 (82)	7 (18)	25 (78%)	7 (22%)	—	—
King et al. (2012)	80.5 (±6.3)	78.4 (±6.5)	72 (77.4)	21 (22.6)	65 (69.9)	28 (31)	63 (67.7%)	47 (50.5%)
Parsons et al. (2012 & 2013)^a^	79.08 (±6.93)	76.90 (±7.61)	77 (71.3)	31 (28.7)	59 (60.8)	38 (39.1)	69 (63.9%)	60 (61.9%)
Lewin et al. (2013)	81.84 (±7.19)	82.73(±7.7)	226 (70.1)	112 (29.9)	242 (64.5)	133 (35.5)	192 (51.2%)	159 (42.4%)
Burton et al. (2013)	80.2 (±6.4)	79.58 (±6.2)	30 (75.0)	10 (25.0)	36 (90.0)	4 (10.0)	24 (60.0%)	27 (67.5%)
Tuntland et al. (2015)	79.9 (±10.4)	78.1 (±9.8)	22 (70.9)	9 (29.0)	19 (63.3)	11 (36.6)	10 (32.3%)	4 (13.3%)
Whitehead et al. (2016)	82.93 (±9.02)	81.93 (±12.96)	11 (73.3)	4 (26.6)	6 (40.0)	9 (60.0)	9 (60.0%)	11 (73.3%)
Hattori et al. (2019)	80.0 (76.3–84.0)	80.0 (76.0–84.0)	131 (68.9)	59 (31.1)	119 (64.3)	66 (35.7)	—	—

^a^ Parsons et al. (2012, 2013) are two separate journals reporting on different outcomes from the same RCT.

### 3.2 Intervention

The intervention, role, components, dose, comparator and service delivery timescale are documented in Table 2.

**TABLE 2:** Intervention components

Study	Intervention	Role	Intervention component	Dose	Comparator	Service timescale
Sheffield et al. (2013)	Ageing in Place: restorative occupational therapy with home care	Occupational Therapists (OTs received enhanced training and supervision)	Adaptative equipment, home modifications, goal setting	4 visits, 9 h, including travel time	Standard Home care	Not reported
King et al. (2012)	Restorative home care	Care Coordinators (Enhanced supervision and training for home care staff)	Goal facilitation. Practice of ADL exercises	1 needs assessment for home care to follow	Standard Home care	Not reported
Parsons et al. (2012, 2013)^a^	Restorative home care	Care coordinator (Gave 2 weeks training and supervision to home care staff)	TARGET goals setting and steps to achieve them	1 needs assessment for home care to follow	Standard Home care	Not reported
Lewin et al. (2013)	HIP: Restorative care	Not stated	Daily living activities (task analysis and redesign, work simplification), equipment (assistive technology), exercise (strength, balance, and endurance)	Min 3 Reablement visits	Standard Home care	12 weeks
Burton et al. (2013)	LIFE exercise programme	Multi‐disciplinary Care Managers Including OTs	Enhanced OTEGO Exercise Programme falls prevention developed in New Zealand (NCOA, 2022) that is combined with everyday ADL activities	3 visits to monitor daily practice	Standard Otego and home care	8 weeks
Tuntland et al. (2015)	Multi‐component rehabilitation	Occupational Therapist (delivery, training, or supervision)	Training in daily activities, adaptations to the environment and activity exercise programmes		Standard Reablement Home care	10 weeks
Whitehead et al. (2016)	OTHERS: Targeted ADL	Occupational Therapist (delivery and weekly progress reviews with Reablement service)	ADL activity's goal setting practising activities, equipment provision and environmental or activity modification	5 × OT visits of 45 mins	Standard Reablement Home care	6 weeks
Hattori et al. (2019)	CoMMIT Reablement	Occupational Therapist (Delivery, training, or supervision) and Rehab Specialist (supervised by OTs)	Motivational interview with participants, goal attainment self‐management skills: to maintain oral health, nutrition, physical activities, activities of daily living and instrumental ADL	2–3 h weekly	Standard Home care	12 weeks

^a^ Parsons et al. (2012, 2013) are two separate journals reporting on different outcomes from the same RCT.

All service models had an element of Reablement home care. However, the service delivery varied, described as including a care coordinator assessment with home carers following a Reablement plan (King et al., 2012; Parsons et al., 2012, 2013), and an assessment followed by face‐to‐face monitoring or review of the person's Reablement progress (Burton et al., 2013; Hattori et al., 2019; Sheffield et al., 2013; Tuntland et al., 2015; Whitehead et al., 2016). Where a study reported a review or monitoring of progress, visits ranged from three to five (Table 2).

The professional role of the Reablement assessor ranged from non‐specified case coordinators (King et al., 2012; Lewin et al., 2013; Parsons et al., 2013), case managers including occupational therapists (Burton et al., 2013) or occupational therapists (Hattori et al., 2019; Sheffield et al., 2013; Tuntland et al., 2015; Whitehead et al., 2016).

The intervention components focused on a complex range of strategies to increase motivation, ability and mobility (Table 2). Functional ability is a descriptive term used for how people complete Activities of Daily Living (ADL): washing, dressing, meal preparation, independent living skills, shopping, and housework. Whereas functional mobility describes the ability to sit to stand, balance and walk.

The service delivery was reported to be achieved in 6 weeks (Whitehead et al., 2016), less than twelve weeks (Hattori et al., 2019; Lewin et al., 2013; Tuntland et al., 2015), or was not specified (King et al., 2012; Parsons et al., 2012; Parsons et al., 2013 & Sheffield et al., 2013).

### 3.3 Control

In all studies, the control group received standard home care. Burton et al. (2013), Tuntland et al. (2015) and Whitehead et al. (2016) used standard Reablement home care as the control. In addition, Burton et al. (2013) gave participants in the control group an exercise information leaflet describing OTEGO Exercise, a fall prevention programme developed in New Zealand (National Council on Ageing (NCOA), 2022).

### 3.4 Outcomes

The outcome measures are tabulated in Appendix S2. Two studies use the dichotomous measure of no ongoing home care service as a primary measure, taken from administrative databases (Hattori et al., 2019; Lewin et al., 2013); three studies report on this outcome as an incidental finding (King et al., 2012; Sheffield et al., 2013; Whitehead et al., 2016).

Two studies used unreferenced, non‐comparable dichotomous ADL measures (Hattori et al., 2019; Lewin et al., 2013). Burton et al. (2013) created a summary measure using combined scores from four outcome measures, valid and reliable when used on their own.  The other studies used outcome measures that gave continuous data to quantify the change in primary or secondary outcomes, these were standardised, valid and reliable.

The effect measures varied; five studies used average difference (95% confidence interval). Lewin et al. (2013) and Hattori et al. (2019) used an odds ratio (95% confidence interval), and Sheffield et al. (2013) used coefficients (standard error).

Table S3, shows the detailed results of each study, and clearly identifyin that the data was heterogenic and justified narrative reporting.

#### 3.4.1 What are the interventions, when are they delivered and by whom?

All studies delivered Reablement interventions with individuals in their homes except Hattori et al. (2019) who delivered some educational aspects to participants in a community group setting.

The interventions investigated were multi‐faceted, with each study focusing on different combinations (Table 2). Sheffield et al. (2013) examined goal setting, adaptative equipment and home modifications. King et al. (2012) examined goal setting and practice of ADL. Lewin et al. (2013) examined ADL, adaptations and exercise. Tuntland et al. (2015) and Whitehead et al. (2016) examined the practise of ADL, equipment provision and environmental modifications. Burton et al. (2013) focused on fall prevention and strength and balance training combined with ADL activities. Parson et al. (2012, 2013) examined participant behaviour change and motivation to attain goals, and Hattori et al. (2019) examined self‐management, behaviour change and motivational coaching for participants.

Lewin et al. (2013) specifically refer to task analysis. Two studies refer to activity modification, a resulting intervention strategy implying analysis has taken place (Tuntland et al., 2015; Whitehead et al., 2016).

Five studies reported that Occupational Therapists were actively involved in the delivery of interventions, training or supervision of home care staff (Table 2). Home Carers and generic Case Managers delivered the intervention in three studies, Table 2 Whitehead et al. (2016) used service evaluation data to evidence delivery of intervention, timing and amount showing an average of five Occupational Therapist visits lasting 45 min.

Three studies repeorted on training or supervision for Home Carer's. King et al. (2012) for Reablement assessment staff and home care coordinators. Tuntland et al. (2015) for all healthcare workers on the ideology of self‐management with therapists providing supervision for Home Carers, focusing on encouraging the participant to exercise and do the daily activities themselves using adaptations to the environment or the activity. Hattori et al. (2019) reported occupational therapists supervised all initial home‐visit assessments and trained the rehabilitation specialists.

#### 3.4.2 How are the effects measured and evaluated?

All studies measured effect by just three primary outcomes: no ongoing service, change to functional ability, and change to the quality of life, except Whitehead et al. (2016) who`s primary outcome was aspects of feasibility. All the studies had secondary outcomes, change to functional ability and functional mobility the most relevant to this review (Appendix S3).

The issue was the diverse range of tools used to measure, some valid and reliable, others not (Appendix S2) and the consequence this had on results.

#### 3.4.3 Are there any long‐term benefits?

Five studies reported a descreased need for ongoing home care post‐intervention (Table 3). The lack of reported data for between group difference and transparency of method, affected the comparison of studies that reported an incidental effect.

**TABLE 3:** Studies that measured the need for reduced home care

Studies that measured the need for reduced home care					
Study	Timing of measure (months/weeks)	Intervention *n* (%)	Control *n* (%)	Between‐group difference	Certainty Grade
Sheffield et al. (2013)	3 m	17/46 (39%)	0/46 (0%)	Not reported	Not Graded
King et al. (2012)	4 m	27/93 (29%)	0/93 (0%)	*p* < 0.001**	Not Graded
Lewin et al. (2013)^a^	3 m	103/375 (27.5%)	238/375 (63.5%)	Odds ratio 0.18 (0.13–0.26) *p* < 0.001**	⨁⨁◯◯Low
Lewin et al. (2013)^a^	12 m	67/375 (17.9%)	151/375 (40.3%)	Odds ratio 0.22 (0.15–0.32) *p* < 0.001**	⨁⨁◯◯ Low
Whitehead et al. (2016)	2 wks.	9/15 (60%)	7/15 (46%)	Not reported	Not Graded
Whitehead et al. (2016)	3 m	13/15 (86%)	9/15 (60%)	Not reported	Not Graded
Whitehead et al. (2016)	6 m	9/15 (60%)	9/15 (60%)	Not reported	Not Graded
Hattori et al. (2019)^a^	4 m	21/190 (11%)	7/185 (3.8%)	Odds ratio 7.3 (2.0–12.5) *p* = 0.007**	⨁⨁⨁◯ Moderate

^a^ Primary outcome measure, **p < 0.05.

**FIGURE 2:**
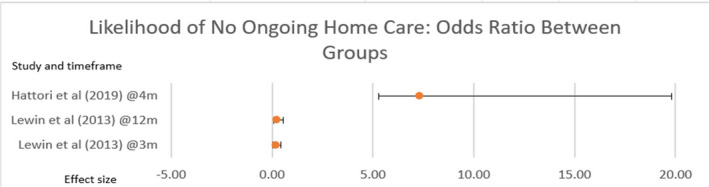
Likelihood of no ongoing home care

Where the need for ongoing home care was a primary outcome measure, effect is presented using odds ratio with 95% confidence intervals, showing a statistical significance (Figure 2).

**FIGURE 3:**
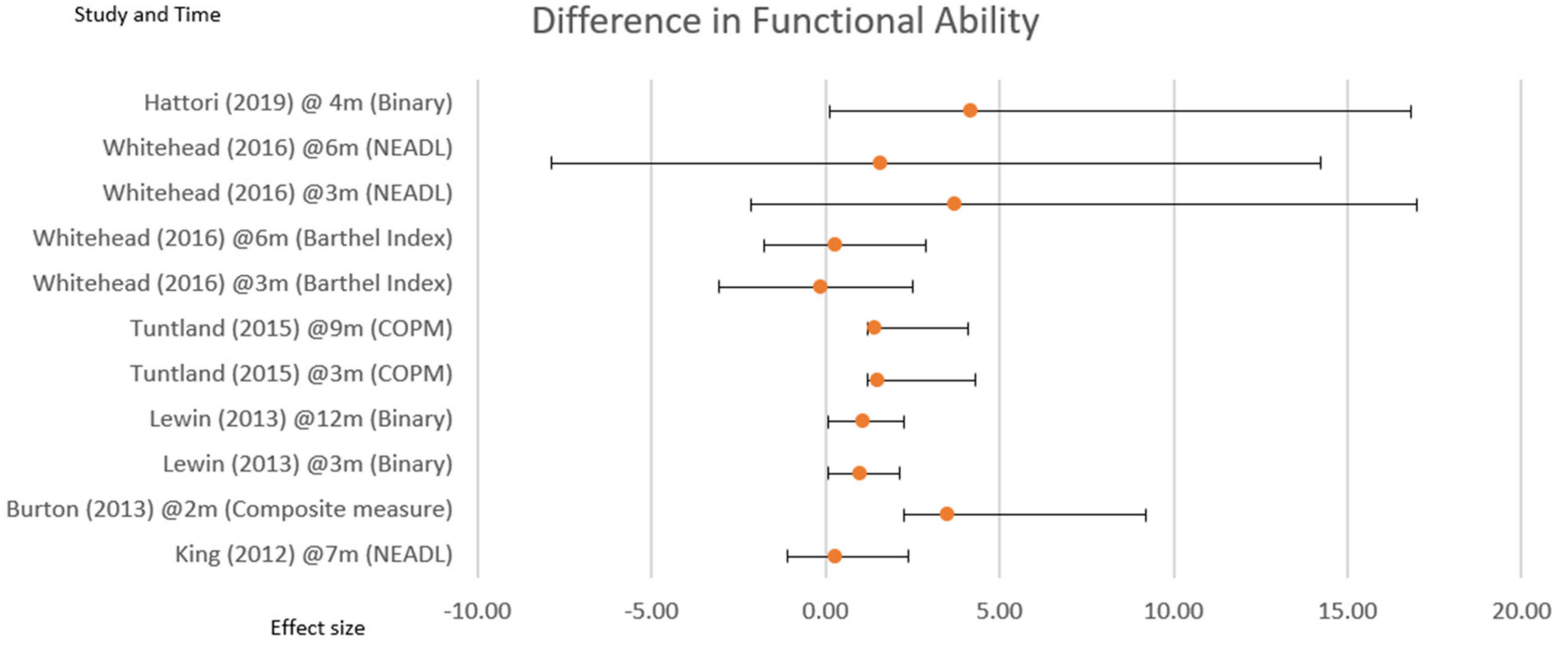
Difference in functional ability

Next, (Table 4) shows the long‐term benefits of functional ability. There is an increase in effect post‐intervention reported in four studies, with a slight decrease after 6 months (Whitehead et al., 2016), and 9 months (Tuntland et al., 2015) (Figure 3).

**TABLE 4:** Change in functional ability

Change in functional ability				
Study	Outcome measure	Timing (months)	Difference between groups (95% confidence interval)	Certainty Grade
Sheffield et al. (2013)	FIM^a^(Mackintosh, 2009)	3	*p* = 0.15**	⨁⨁⨁◯ Moderate
King et al. (2012)	NEADL (Nouri & Lincoln, 1987)	7	0.3 (−1.4 to 2.1)	⨁⨁⨁⨁ High
Burton et al. (2013)	Composite measure^a^	2	3.5 (1.25–5.70) and *p* = 0.003**	⨁⨁⨁◯ Moderate
Lewin et al. (2013)	Binary Scale (not referenced)	3	Odds ratio 1.02 (0.95– 1.09) *p* = 0.529**	⨁⨁◯◯ Low
Lewin et al. (2013)	Binary Scale (not referenced)	12	Odds Ratio 1.08 (1.00–1.17) *p* = 0.048**	⨁⨁◯◯ Low
Tuntland et al. (2015)	COPM^a^ (Carswell et al., 2004)	3	1.5 (0.3–2.8) *p* = 0.02**	⨁⨁⨁⨁ High
Tuntland et al. (2015)	COPM^a^(Carswell et al., 2004)	9	1.4 (0.2–2.7) *p* = 0.03**	⨁⨁⨁⨁ High
Whitehead et al. (2016)	Barthel Index (Colin et al., 1988)	3	−0.13 (1.33) (CI −2.91 to 2.65)	⨁⨁⨁⨁ High
Whitehead et al. (2016)	Barthel Index (Colin et al., 1988)	6	0.28 (1.12) −2.06 to 2.61	⨁⨁⨁⨁ High
Whitehead et al. (2016)	NEADL (Nouri & Lincoln, 1987)	3	3.72 (4.58) (−5.85 to 13.27)	⨁⨁⨁⨁ High
Whitehead et al. (2016)	NEADL (Nouri & Lincoln, 1987)	6	1.58 (5.28) (19.47 to 12.64)	⨁⨁⨁⨁ High
Hattori et al. (2019)	Binary Scale (not referenced)	4	ind = 4.2% (−4.1 to 12.6) dep =13.9% (4.0 to 23.7) dep in 2 or more = 4.3% (−4.0 to 12.6)	⨁⨁⨁◯ Moderate

^a^ Primary outcome measure, **p < 0.5.

Comparing each study reporting a positive effect, Sheffield et al. (2013) measures with the Functional Independence Measure (Mackintosh, 2009), reporting statistical effect as a p value but with no further between‐group comparable data. Next, King et al. (2012) use Nottingham Extended Activities of Daily Living (NEADL) (Nouri & Lincoln, 1987), measuring the between‐arm mean difference in score, from baseline to 7 months as 0.3 (−1.4 to 2.1), *p* = 0.71.

The third study (Tuntland et al., 2015), uses the Canadian Occupational Performance Measure (COPM) (Carswell et al., 2004), reporting a statistically significant self‐perceived mean difference at 3 months, 1.5 (0.3–2.8) *p* = 0.02. In the same study, at 9 months, the results show the mean difference between interventions is 1.4 (0.2–2.7) *p* = 0.03.

Whereas (Whitehead et al., 2016) the fourth study, uses NEADL (Nouri & Lincoln, 1987), and median average difference with the inter‐quartile range due to the small sample of *n* = 30. At 3 months, the difference in functional ability is 3.72 (4.58) with a 95% confidence interval (−5.83 to 13.27) and at 6 months, 1.58 (5.28) with a 95% confidence interval (−9.47 to 12.64).

Next, taking change in the quality of life, four studies evidenced improvement (Table 5) Tuntland et al. (2015) used COOP/Wonka score (Kinnersley et al., 1995), whereas other studies used the Short‐Form SF‐36 health survey (Ware et al., 2000), reporting effect based on the total score for SF‐36, combining the physical and mental aspect.

**FIGURE 4:**
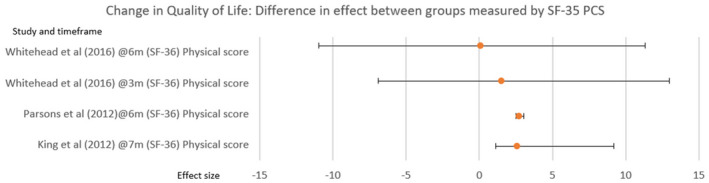
Change in quality of life

Comparisons between studies measuring quality of life using SF‐36 (Figure 4) show a similar difference in effect for the SF‐36 physical score at 6–7 months; King et al. (2012) and Parsons et al. (2012) used a similar sample size. Whitehead et al. (2016) report less effect is reported at both 3 and 6 months with a smaller sample.

**TABLE 5:** Change in quality of life

Change in quality of life: Difference in effect between groups			
Study	Timing (months)	Outcome measure	Average difference between groups *n* (95% confidence interval)
King et al. (2012)	7	(SF‐36) Physical score (Ware et al., 2000)	2.6 (−1.5 to 6.6) *p* = 0.22*
Parsons et al. (2012)	6	(SF‐36) Physical score (Ware et al., 2000)	2.7 (−0.2 to 0.35) *p* = 0.0002*
Tuntland et al. (2015)	3	COOP/Wonca Score (Kinnersley et al., 1995)	−0.4 (−0.9 to 0.2) *p* = 0.21*
Tuntland et al. (2015)	6	COOP/Wonca Score (Kinnersley et al., 1995)	−0.4 (−0.3–0.5) *p* = 0.22*
Whitehead et al. (2016)	3	(SF‐36) Physical score (Ware et al., 2000)	1.52 (4.75) (−8.43 to 11.47)
Whitehead et al. (2016)	6	(SF‐36) Physical score (Ware et al., 2000)	0.09 (5.33) (−11.06 to 11.24)

* *p* < 0.5.

Lastly, three studies report the positive effect on functional mobility, Table 6. Burton et al. (2013), did not achieve their primary outcome using a composite of valid and reliable outcome measures, aiming to evidence the statistical effect of LIFE interventions in a summary variable at 2 months, (Table 6).

**FIGURE 5:**
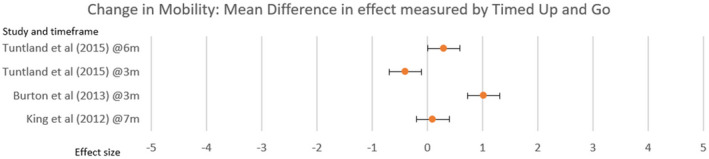
Change in mobility

King et al. (2012) and Tuntland et al. (2015) used The Timed Up and Go, a valid and reliable outcome measure (Ashley et al., 2019), as a secondary outcome measure, Figure 5. Despite a positive effect, the studies using this measure were unable to produce a statistically significant short‐ or long‐term result for changes to functional mobility.

**TABLE 6:** Change in mobility

Change in mobility			
Study	Timing (months)	Measure	Average difference between groups (95% confidence interval)
King et al. (2012)	7	Timed up and go (Podsiadlo & Richardson, 1991)	0.1 (−4.2 to 4.1), *p* = 0.98
Burton et al. (2013)	3	Timed up and go (Podsiadlo & Richardson, 1991)	1.02 (−4.86 to 2.83), *p* = 0.983
Tuntland et al. (2015)	3	Timed up and go (Podsiadlo & Richardson, 1991)	−0.4 (−4.4 to 3.5), *p* = 0.82
Tuntland et al. (2015)	6	Timed up and go (Podsiadlo & Richardson, 1991)	0.3 (−3.7 to 4.3), *p* = 0.88

### 3.5 Risk of bias

Overall, four studies scored low risk of bias, three studies had some risk of bias, and two studies had a high risk of bias (Table 7).

**TABLE 7:** Study overall risk of bias assessment

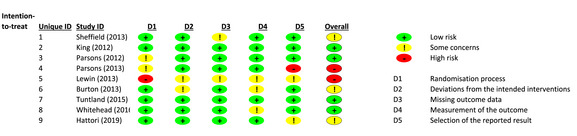

The reviewers considered each risk domain for each outcome. There was no identified risk of bias in any domain for Tuntland et al. (2015), or King et al. (2012) who justified using a cluster design to facilitate the staggered paid caregivers training, avoiding contamination between the groups.

In Parsons et al. (2012), the randomisation was a concern because there was little detail on the process reported, and the reviewers took the study on face value and assumed integrity, assessing as low risk overall.

Whitehead et al. (2016) used valid and reliable standardised outcome measures. Howevever the reviewers were concerned about missed data or misinterpretaion of data, the study did not identify this limitation. Despite blinding the occupational therapy assessor, having unblinded participants and home care staff meant the assessor could have guessed the allocation group. When the reviewers considered collection of outcome data was face‐to‐face in the participant's home, and immediately entered into a database, the reviewers agreed a judgement that any bias through missed data or misinterpretation of data was reduced, and reduce the overall risk of bias score.

The reviewers upheld their concerns about the five RCTs with an overall risk of bias. In Sheffield et al. (2013) it was in the missing outcome domain, due to a high attrition rate explained as ‘age ineligibility’, those participants who dropped out had higher dependency scores on the Functional Independence Measure. Burton et al. (2013) deviated from intended interventions and outcome measurement. Hattori et al. (2019), had selective reporting bias because the non‐significant results were reported in online supplementary files, and the referenced protocol detailing the analysis plan were unobtainable despite an in‐depth online search and email correspondence to the author.

The reviewers judged Lewin et al. (2013), and Parsons et al. (2013), to have high‐risk concerns overall. In Lewin et al. (2013) study, it was in randomisation, and selective reporting due to the lack of operator blinding in the electronic allocation sequence, and the participant baseline differences favouring the intervention.

Bias in Parsons et al. (2013) study was due to reported baseline imbalances weighted towards the intervention group, suggesting recruitment bias. For the secondary outcomes, Parsons et al (2013) only reported functional mobility and social support, despite the intervention focusing on goal attainment. Given that the TARGET intervention focused on person‐centred goal setting and an ADL outcome measure is used at baseline, this raised a concern about reporting bias based on favourable results.

### 3.6 Certainty of evidence

The reviewers quality assured the RCTs to determine certainty of effect, based on the assessment of five domains: risk of bias, inconsistency, indirectness, imprecision and publication bias (Schünemann et al., 2020), There were five studies with risk of bias limitations in either design or execution of the method (Table 7).

There was no inconsistency in any study serious enough to downgrade the evidence. In all studies reviewed, the evidence directly answered the review question, and there were no concerns about indirectness. In all the studies, the reported results were precise, and the quality of evidence was upheld (Appendix S3).

There was a probability of publication bias in Parsons et al. (2013), both reviewers agreed the reporting bias was not serious enough to downgrade the quality of evidence.

The reviewers considered the magnitude of the effect, in the case of Lewin et al. (2013) study they deemed it worthy of an upgrade to moderate certainty because of the large relative effect at three months with a large sample, a low odds ratio with a narrow confidence interval, and statistically significant result.

## 4 DISCUSSION

The purpose of this systematic review, was to identify, describe, and critically appraise the interventions delivered and their effects on outcomes of Reablement using robust methodology; eight RCTs met the eligibility criteria. The reviewers found the data was methodologically heterogenic.

The search criteria for this review were based on evidenced based reasoning that Reablement interventions should continue until people have maximised their abilities and reached their person‐centered goals; time‐limited but not time‐restricted (Clotworthy et al., 2021; SCIE, 2020; Doh, 2020). Unlike previous reviews of RCTs, the reviewers placed no restrictions on intervention delivery timescale in the eligibility criteria.

This review challenges a historical systematic review of RCTs that found no evidence of effect for Reablement delivered in six weeks (Legg et al., 2015), strengthens Cochrane et al. (2016) who found low‐quality evidence for interventions delivered up to twelve weeks, and supports Sims‐Gould et al. (2017) who found limited generalisability of results for interventions delivered under six months.

To enable the optimum content and configuration of Reablement interventions (Boniface et al., 2013), interventions must be person centered and delivered flexibly. This review grouped the multi faceted interventions, examining their effect on functional ability, functional mobility, and quality of life outcome measures. The only study that attempted to change behaviour in a group setting (Hattori et al., 2019), had selective reporting bias weakening the overall certainty of the results.

The outcome measurement tools for functional ability were incomparable, with exception of two studies that used NEADL (Nouri & Lincoln, 1987), fig 3. Tuntland et al. (2015) used COPM (Carswell et al., 2004) to measure self perceived improvement in ADL, use of a comparable assessor administered measure would have strengthened their study. Likewise, studies considering functional ADL could strengthened results using a persons self perceived measure. One study (Burton, 2013), attempted to use a summary measure to determine improved function, this was confusing and the number of measures extensive. The reviewers found there was a risk of bias in this study due to deviations from intended interventions, and they graded the overall certainty of evidence as moderate. The unreferenced dichotomous measure used by Hattori et al. (2019) to measure functional ability, was incomparable with Lewin et al. (2013), neither clearly indicate high or low score as most effective, meaning comparison was not possible.

This review found improved effect for increased functional ability in five studies, table 4: fig 3. This was statistically significant in four of the studies, adding to the evidence base that interventions targeting ADL reduce dependency.

There was homogony for improved effect in mobility, fig 5 using the timed up and go outcome measure, but no studies were statistically significant.

There was homogony in the quality‐of‐life effect, measured using the health‐related SF‐36, fig 4. However, Parsons et al. (2012) used the SF‐36 as a primary outcome measure for the effectiveness of goal setting. Using quality of life to measure clinical intervention effect is limiting because it is a personal construct, less accurate and responsive than specific outcome measures in situations where interventions aim to achieve a particular outcome (Higginson & Carr, 2001).

Furthermore, the method for calculating the SF‐36 was unclear in both King et al. (2012) and Parsons et al. (2012). A scoping review of studies reporting a total quality of life score using SF‐36, evidenced 129 (75.0%) of the 172 studies did not specify the method for calculating the SF‐36 total score (Lins & Carvalho, 2016). The reviewers compared the separate results of the SF‐36 physical (PCS) and mental (MCS) scores (RAND, 2021). The improved effect of quality‐of‐life outcomes measured using SF‐36 (PCS) were statistically significant in three studies (fig 4), suggesting that Reablement interventions have a positive impact for people.

The heterogeneity in outcome measures has implications for comparison in systematic reviews, and choice of outcome measures can be a strength or limitation to the study. Beresford et al. (2019) established a requirement for a range of outcomes, including self reports and mental health outcomes, measured over an extended time. The reviewers go further, arguing that a preferred homogeneous outcome measure for Reablement interventions would enable comparison of effect. This should include functional ability, mobility, and quality of life. Until then, it would better to determine the benefits of Reablement using other methodology.

The professional role of the person delivering interventions was unclear in studies with a non‐specific care coordinator, table 2. Despite a positive effect on the requirement for ongoing services in the studies with occupational therapy involvement at three months (Sheffield et al., 2013; Tuntland et al., 2015; Whitehead et al., 2016) and at 4 months Hattori et al. (2019), it wasn`t possible to unequivocally determine whether professional role influenced the difference in outcome due to heterogenetic data. The only UK study had a small sample n=30 (Whitehead et al., 2016), limiting the results for an otherwise methodologically sound study. The primary outcome was to explore feasibility of design, and whilst the study evidenced the number of visits by role, it did not explore whether frequency of Occupational Therapist visits optimised the outcomes.

Pettersson & Iwarsson, (2017) identified in their literature review that there was a lack of definition of interventions and professional roles, and this remains a problem for evaluating Reablement intervention outcomes in relation to economic effectiveness and quality assurance. Reablement services deliver interventions for people with varying degrees of complexity and need, and the cost of regulated specialist professionals is greater than unqualified workers, therefore the staffing role and responsibility should be clear in any research examining the effectiveness of Reablement outcomes.

Three studies described Reablement workers competency to deliver interventions, their supervision, and training (King et al., 2012; Tuntland et al., 2015; Hattori et al., 2019). They do not specify how these can influence or assure better outcomes (Sims‐Gould et al., 2017). This lack of detail evidenced a need for further research on Reablement workers competency, training, and supervision to enable a greater understanding of Reablement as a complex intervention. Furthermore, occupational therapists have a role in training Reablement workers to operate in an enabling way (Dibsdall, 2021).

Whilst the evidence remains thin on the effectiveness of Reablement, this review critically examines the types of intervention, outcomes, effect, professional role, providing new understanding of Reablement. The reviewers ascertained the need for clearly defined roles for assessment, delivery, and review. The impact of training Home Carers on the Reablement ethos, in relation to intervention effect outcomes is a recommendation for future research (Dibsdall, 2019; Rooijackers et al., 2021).

## 5 STRENGTHS AND LIMITATIONS OF THE INCLUDED STUDIES

The intervention effect was positive in all studies. Howevever, the outcome measures were diverse, and comparison of heterogeneous outcome data would be misleading. This limited the extent the reviewere could accurately compare these RCTs.

Overall, five studies had a risk of bias limitations in either design or method (Table 7), two large sample RCTs were judged high risk overall. (Lewin et al., 2013; Parsons et al., 2013). While the larger samples strengthen study results, these limitations affected the reviewers confidence in the findings.

Certainty of evidence was graded high in four studies for the primary outcome (King et al., 2012; Parsons et al., 2012; Tuntland et al., 2015; Whitehead et al., 2016) and moderate in three (Burton et al., 2013; Hattori et al., 2019; Lewin et al., 2013). The secondary outcomes reported by Parsons et al. (2013) in a separate journal article for the same RCT were graded as moderate certainty.

### 5.1 Strengths and limitations of the review method

A strength of this systematic review is the use of reliable methodology (Page et al; 2020). The reviewers recorded on a piloted data recording form (Li, Higgins & Deeks, 2020) enabling challenge of preconceptions or selection bias; this review is reproduceable. The use of a protocol, a review strategy implimented by two researchers from different professional backgrounds, the RoB2 risk of bias framework (Cochrane, 2021), GRADE (Hultcrantz et al, 2017) to determine the certainty of the evidence (Boutron et al., 2020), and reflexivity to reduce professional bias gave confidence in the review findings.

The reviewers limited the search criteria to peer‐reviewed RCTs, this excluded other relevant experimental studies, creating a narrower review (Schünemann et al., 2020), limiting the findings. The reviewers agreed to include a feasibility RCT (Whitehead et al., 2016) and prospective RCT (Parsons et al., 2012) as they met the search eligibility criteria. The search terms were not extensive and this was a limitation. The reviewers excluded studies with participants not in receipt of a home care service, a limiting factor as informal care givers deliver home care for cultural or financial reasons.

The synthesis method clearly identified data from the reviewed studies was heterogenic, the variety of outcome measures used gave no scope for meta‐analysis, subgroup analysis or meta‐regression, limiting the extent of comparison. The SWiM reporting guideline (Campbell et al., 2020) strengthened the narrative reporting method. The use of GRADE (Hultcrantz et al., 2017) addressed the subjectiveness of the reviewers analyisis of the quality of the studies, strengthening the assessment of certainty in the effect.

The method gave an opportunity to consider confidence in effects, quality, similarities, the impact of bias and applicability of the findings to the research question. The reviewers used the method to assess whether the effectiveness of the interventions was sensitive to clinical or methodological heterogeneity and whether the intervention effect itself was enough to eliminate any risk in the bias domains; this sensitivity analysis ensures confidence in the intervention effect, strengthening the study (Deeks et al., 2020).

### 5.2 Can the findings be generalised?

The intervention delivery, amount and type of interventions described in all studies could not be generalised due to the methodological heterogeneity of the data. The reviewers evidenced external validity in two studies with unrestricted age samples (Tuntland et al., 2015; Whitehead et al., 2016). The only health condition exclusions, dementia and palliative care suggest that Reablement has efficacy in a diverse range of conditions. Internationally, Health and Social Care organisations, policy drivers, workforce mix, and population culture vary, limiting generalisability.

It was not possible to determine whether a professional role has a statistical consequence that could be generalised. A definitive large sample UK trial aiming to determine the effect of Reablement interventions and the professional role administering them is required. The ethical and methodological considerations of RCTs are complex to resolve in a Social Care setting: randomising to intervention; blinding the researcher, professional completing the assessment and review, Reablement workers delivering the intervention and the participant receiving the intervention, meaning that it is most appropriate to use a cohort study method.

## 6 CONCLUSIONS

The reviewers found diversity in the outcome measure indicating future research should establish an agreed outcome measure for Reablement, including need for ongoing home care, functional ability, mobility, and quality of life, to evidence Reablements effect.

The results show the need for an ongoing home care service decreased in five studies Table 3: Figure 2, with improved effect at three months shown in studies with occupational therapist involvement. Functional ability increased statistically in four studies at three months, Table 2. An increase in quality of life was statistically significant in three studies at six and seven months, table 5. None of the studies reported a statistically significant improvement in functional mobility, Table 6. The outcome of Reablement was most beneficial at three to four months, with some sustainability beyond six months (Tuntland et al., 2015; Whitehead et al., 2016).

Studies with Occupational Therapist's involvement showed a greater effect on outcomes, this was not statistically significantTherefore, the effect of professional role delivering Reabelement could not be generalised. Furthermore, there was scant consideration of competency or training for the Reablement worker delivering the interventions and whether this can improve outcomes, this warrants further research.

The reviewed RCTs evidenced clinical feasibility and appropriateness (Robertson‐Malt, 2014), multi‐faceted Reablement interventions are effective in the context of Health and Social Care. A large sample UK trial aiming to determine the effect of Reablement interventions, and who is best placed to deliver them, is necessary.

## CONFLICT OF INTEREST

There is no conflict of interest, the views expressed are those of the author and not necessarily those of the NIHR, Nottinghamshire County Council or the Department of Health and Social Care.

## Supporting information


**Appendix S1:** **Appendix S2:**
Appendix S3:

## Data Availability

The data that support the findings of this study are available from the corresponding author upon reasonable request.
