# Supplementary material for: An investigation of Reablement or restorative homecare interventions and outcome effects: A systematic review of randomised control trials
Source: Health Soc Care Community. 2022 Dec 2;30(6):e6586–600. doi: 10.1111/hsc.14108 (PMC10107871; doi:10.1111/hsc.14108)
Supplement: Supplementary file 1 — Appendix S1: Appendix S2: Appendix S3: [file HSC-30-e6586-s001.docx]

**Supplementary Appendix**

# Supplementary Appendix 1: Included and excluded studies with summary of reasons

The included studies are highlighted green and the excluded studies red, those highlighted in yellow are published protocols for RCT, that might meet the inclusion criteria once study results are published.

| Author and Year | include | RCT | Consenting  Adult Over 18 | **Receiving**  **Homecare**  **service** | Occupational therapy  interventions | Intervention delivered in  Persons Home setting | Diagnosis of dementia | Diagnosis of Palliative or end of life |
| --- | --- | --- | --- | --- | --- | --- | --- | --- |
| Stewart (2005) | no | yes | yes | no | yes | yes | not reported | not reported |
| Gitlin (2006) | no | yes | yes | no | yes | yes | yes (mms* >24) | no |
| Gitlin (2009) | no | yes | yes | no | yes | yes | yes (mms>23) | not reported |
| Claire (2009) | no | yes | yes | no | yes (great cognitive rehabilitation) | yes | yes (mms >18) | no |
| Gitlin (2010) | no | yes | yes | no | yes | yes | yes(mms >24) | no |
| Page (2011) | no | yes | yes | no | yes (ADL mental practice) | yes | no | no |
| Voigt-Radloff (2011) | no | yes | yes | no | yes | yes | yes  (mms 14 to 24) | no |
| Sheffield (2012) | no | yes | yes | yes | yes (aging in place) | yes | no | no |
| King (2012) | yes | yes | yes | yes | yes (goal setting and ADL practice) | yes | no | no |
| Parsons (2013) | yes | yes | yes | yes | yes (target goal setting) | yes | no | no |
| Lewin (2013) | yes | yes | yes | yes | yes (hip) | yes | no | no |
| Burton (2013) | yes | yes | yes | yes | yes (life adl exercises) | yes | no | no |
| Cameron (2013) | no | yes | yes | no | no (frailty) | yes | no | no |
| Senior (2014) | no | yes | yes | yes | no (case management and restorative home care) | no | no | not reported |
| Tuntland (2015) | yes | yes | yes | yes | yes | yes | no | no |
| Fairhall (2015) | no | yes | yes | no | no | yes | no | no |
| Whitehead (2016) | yes | yes | yes | yes | yes | yes | no | no |
| Parsons (2017) | no | yes | no  (proxy consent by next of kin) | yes | no (comprehensive geriatric assessment with goals) | yes | yes | no |
| Kessler (2017) | no | yes | yes | no (6% receiving home care) | yes (coaching) | yes | no | not reported |
| Schwartz (2017) | no | yes | yes | no | no (medication) | yes | not reported | not reported |
| Chiung-ju (2017) | no | yes | yes | no | yes (3 step ADL workout) | yes | no | no |
| Yoshida (2018) | no | yes | yes | no | yes(skill balance) | no | no | no |
| Metzelthin (2018) | no | RCT protocol | yes | yes | yes (stay active at home) | yes | no | no |
| Szanton (2019) | no | yes | yes | no | yes (capable) | yes | no | No |
| Hattori (2019) | yes | yes | yes | yes | yes (commit) | yes | no | no |
| Bergostrom (2019) | no | feasibility for RCT | yes | yes | yes(assist1:0) | yes | no | no |
| Han (2020) | yes | yes | yes | no | yes | yes | no | no |
| Jeon (2020) | no | yes | yes | yes | yes | yes | yes (mms 16 to 30) | no |

*mms= Mini Mental Score 0 to 17 is severe cognitive impairment

# Supplementary Appendix 2: Outcomes and outcome measures

| Study | Outcomes (Primary or Secondary) | Outcome measure | Type | Follow up |
| --- | --- | --- | --- | --- |
| Sheffield et al  (2012) | Primary:  Change in 4 measures   - Home safety - Functional ability - Fear of falling - Quality of life | SAFER-HOME (score range 0-222) [Chiu &Oliver, 2006]  Functional Independence Measure (FIM) (score range 0-140) [Mackintosh, 2009]  Short Falls Efficacy Scale (FES) (score range 0-280 [Kempen et al,2008]  EQ5D (score range 0-5) [Barton et al, 2007] | C* | Baseline &  3 months |
| King et al (2012) | Primary:  Change in health-related quality of life | Short Form 36 (SF36) Health Survey [Ware et al, 2000] | C* | Baseline, 4 & 7 months |
|  | Secondary:  Functional ability | Nottingham Extended ADL (ordinal binary measure score 0-66 high score =greater independence [Nouri &Lincoln, 1987] | C* | Baseline, 4 & 7 months |
| Parsons et al (2012)  Parsons et al (2013) | Primary:  Health related quality of life | Short-Form 36 Health Survey [Ware et al, 2000] | C* | Baseline &  6 months |
|  | Secondary:  Change in participants’ physical function over time | Short physical performance battery [Guralnik et al,1994] | O* | Baseline &  6 months |
|  | Secondary:  Change in social support | Dukes social support index [Koenig, 1993] | O* | Baseline &  6 months |
| Lewin et al (2013) | Primary:  No ongoing personal care services at 3 months and 12 months | Binary measure | D* | Baseline, 3 & 12 months |
|  | Secondary:  Functional Status ADL and IADL | Binary measure (not referenced)  (lower ADL score= higher dependency) | D* | Baseline, 3 & 12 months |
| Burton et al (2013) | Primary:  Change to Functional ability | Composite measure: incorporating balance, strength, mobility summerised by measuring functional reach, Chair Sit to Stand, Tandem walk [Steffan et al,2002] and Timed Up and Go [Podsiadlo & Richardson, 1991] | C* | Baseline &  8 weeks |
|  | Secondary:  Level of function and disability in everyday activities. | The Late Life Function and Disability instruments [Haley et al, 2002] | C* | Baseline &  8 weeks |
| Tuntland et al (2015) | Primary:  Change to self-rated perceived performance in self-care, productivity and leisure. | Canadian Occupational Performance Measure (COPM) [Carswell et al, 2004] | C* | Baseline, 3 & 9 months |
|  | Secondary:  Functional Mobility | Timed up and go [Podsiadlo & Richardson, 1991] | C* | Baseline, 3 & 9 months |
|  | Secondary:  Health related quality of life | COOP/Wonka (revised version) [Kinnersley et al 1995] | C* | Baseline, 3 & 9 months |
|  | Secondary:  Grip strength | Jamar Dynamometer [Bohannon et al, 2007] | C* | Baseline, 3 & 9 months |
| Whitehead (2016) | Primary:  Aspects of feasibility | Eligibility, recruitment, intervention delivery, attrition and suitability and sensitivity of outcome measures | C* | 2 weeks, 3 & 6 months |
|  | Secondary: Independence in Activities of Daily Living | Barthel Index [Colin et al, 1988] | C* | 2 weeks, 3 & 6 months |
|  |  | Nottingham Extended Activities of Daily Living [Nouri &Lincoln, 1987] | C* | 2 weeks, 3 & 6 months |
|  | Secondary:  Health-related and social care-related quality of life | EQ5D [Barton et al, 2007] | C* | 2 weeks, 3 & 6 months |
|  |  | ASCOT [PSSRU, 2018] | C* | 2 weeks, 3 & 6 months |
|  |  | SF-36 PCS [Ware et al, 2000]  SF-36 MCS [Ware et al, 2000] | C* | 2 weeks, 3 & 6 months |
| Hattori (2019) | Primary:  No long-term personal care services required | Binary measure | D* | Baseline, 4 & 7 months |
|  | Secondary: Functional ADL | Difference in ADL and IADL (not referenced) | D* | Baseline, 4 & 7 months |
|  | Secondary:  Non adverse risk | Hospitalisations or death | D* | Baseline, 4 & 7 months |

*C= continuous, D=dichotomous, O=ordinal

## Supplementary Appendix 3: Primary and Secondary Outcome Results

| **Primary Outcomes** | | | | | | | |
| --- | --- | --- | --- | --- | --- | --- | --- |
| **Outcome** | **study** | **n=** | **t=** | **Illustrative comparative risks (95% CI)** | | **Relative effect:**  **odds ratio (95% CI) and p value** | **Certainty of evidence (GRADE)*** |
|  |  |  |  | Assumed risk (intervention) | Corresponding risk (control) |  |  |
| No ongoing care  services | Lewin et al (2013) | 750 | 3m | 103/375 (27.5%) | 238/375 (63.5%) | Odds ratio 1.18 (0.13 to 0.26) p<0.001 | ⨁⨁◯◯ LOW upgraded to  ⨁⨁⨁◯ MODERATE |
|  |  | 750 | 12m | 67/375 (17.9%) | 151/375 (40.3%) | Odds ratio 0.22 (0.15 to 0.32) p<0.001 | ⨁⨁◯◯ LOW  upgraded to  ⨁⨁⨁◯ MODERATE |
| No ongoing care  services | Hattori et al (2019) | 375 | 4m | 21/190 (11%) | 7/185 (3.8%) | Odds ratio 7.3 (2.0 to 12.5)  p=0.007 | ⨁⨁⨁◯ MODERATE |
| Change in functional ability | Sheffield et al  (2012) | 90 | 3m | Coefficient (standard error)  -2.14 (2.03) | Constant Coefficient (standard error)  28.44 (8.76) | P=0.15 | ⨁⨁⨁◯ MODERATE |
| Change in functional ability | Burton et al (2013) | 80 | 2m | Mean (SD)  7.51 (5.98) | Mean (SD)  4.04 (3.32) | 95% confidence interval  3.5 (1.25 to 5.70) and p=0.003. | ⨁⨁⨁◯ MODERATE |
| Change in functional ability | Tuntland et al  (2015) | 61 | 3m | Mean (95% CI)  6.9/31(6.1 to 7.8)  *COPM Scale 1 to 10 (highest best) | Mean (95% CI)  5.5/30 (4.7 to 6.3) | Mean difference (95%CI)  1.5 (0.3 to 2.8) p= 0.02  Adjusted effect size=  0.8 | ⨁⨁⨁⨁ HIGH |
|  |  |  | 9m | Mean (95%CI)  6.3 (5.0 to 7.6)  *COPM Scale 1 to 10 (highest best) | Mean (95%CI)  4.8 (4.1 to5.5) | Mean difference (95%CI)  1.4 (95%CI 0.2 to 2.7) p= 0.03  Adjusted effect size=0.7 | ⨁⨁⨁⨁ HIGH |
| Change in health-related quality of life | Sheffield et al  (2012) | 90 | 3m | Coefficient (standard error)  0.08 (0.04)  * EQ5D | Constant Coefficient (standard error)  0.19 (0.10) | P=0.3 | ⨁⨁⨁◯ MODERATE |
| Change in health-related quality of life | King et al (2012) | 186 | 4m | Mean (SE) Total:57.8 (1.2)  SF36 PCS:  46.3 (1.2)  SF36MCS:  65.7 (1.3) | Mean (SE) Total:  57.6 (1.5)  SF36 PCS:  45.9 (1.6)  SF36MCS:  65.7 (1.5) | NOT REPORTED | ⨁⨁⨁⨁ HIGH |
|  |  |  | 7m | Mean (SE)  Total: 59.5 (1.4)  SF36 PCS:  46.4 (1.5)  SF36MCS:  67.7 (1.4) | Mean (SE)  Total: 57.7 (1.4)  SF36 PCS:  45.4 (1.5)  SF36MCS:  66.4 (1.4) | Mean difference (95%CI) and p value  Total: 3.8 (0.0 to 7.7)  P=0.05  SF36 PCS:  2.6 (-1.5 to 6.6) P=0.22  SF36 MCS:  4.2 (0.0 to 8.4) P=0.05 | ⨁⨁⨁⨁ HIGH |
| Change in health-related quality of life | Parsons et al (2012) | 205 | 6m | Least mean squared (SE)  Total: 61.92 (3.32)  SF36 PCS:  54.04 (3.52)  SF36MCS:  63.46 (3.31) | Least mean squared (SE)  Total:55.45 (3.10)  SF36 PCS:  51.31 (3.42)  SF36MCS:  58.52 (3.10) | P value  Total <0.0001  SF36 PCS:  p=0.0002  SF36MCS:  p=0.0003 | ⨁⨁⨁⨁ HIGH |
| Change in functional mobility | Burton et al (2013) | 80 | 2m | Mean (SD  -0.55 (0.53) | Mean (SD)  -0.29 (0.68) | 95% confidence interval  (-0.56 to -0.03)  P=0.083 | ⨁⨁⨁◯ MODERATE |
| Change in fear of falling | Sheffield et al  (2012) | 90 | 3m | Coefficient (SE)  -2.22 (1.29)  *FES | Constant Coefficient (SE)  7.13 (3.30) | P value  P<0.05 | ⨁⨁⨁◯ MODERATE |
| Change in  Education | Sheffield et al  (2012) |  |  | Coefficient (SE)  -15.87 (2.07)  *SAFER-HOME | Constant Coefficient (SE)  4.49 (5.18) | P value  P<0.0005 | ⨁⨁⨁◯ MODERATE |
| Primary: Aspects of feasibility | Whitehead et al (2016) | 30 | 3m |  |  | Average  OT Visit = 5  Average time= 45 mins.  50/106 met the eligibility criteria. consent rate was 60% of those eligible. | Not graded as a feasibility of design outcome |
| **Secondary Outcomes** | | | | | | | |
| **Outcome** | **study** | **n=** | **t=** | **Illustrative comparative risks (95% CI)** | | **Relative effect:**  **odds ratio (95% CI) and p value** | **Certainty of evidence (GRADE)*** |
|  |  |  |  | Assumed risk (intervention) | Corresponding risk (control) |  |  |
| Change in functional  ability | King et al (2012) | 186 | 4m | Mean (SE)  44.3/93 (0.9) | Mean (SE)  45 /93 (1.0) | NOT REPORTED | Not assessed |
|  |  | 186 | 7m | Mean (SE)  44.2/93 (1.0) | Mean (SE)  44.4/93 (1.1) | Difference (SE) (95%CI) and p value  0.3 (-1.4 to 2.1) | ⨁⨁⨁⨁ HIGH |
| Change in functional  ability | Lewin et al (2013) | 150 | 3m | Not reported | Not reported | Odds ratio 1.02 (0.95 to 1.09) P=0.529 | ⨁⨁◯◯ LOW |
|  |  | 150 | 12m | n=100 (%)  ADL and IADL functional outcome results based on completed data: * not intent to treat analysis | n= 98(%) | Odds Ratio 1.08 (1.00 to 1.17) p=0.048 | ⨁⨁◯◯ LOW |
| Change in functional  ability | Whitehead et al (2016) | 30 | 3m | Median (SE)  Not reported | Median (SE)  Not reported | Median difference (SE) (95% CI)  3.72 (4.58) (CI−5.83 to 13.27) *NEADL scale: 0–66 (highest best)  −0.13 (1.33) (CI −2.91 to 2.65) *Barthel index scale 0-20(highest best) | ⨁⨁⨁⨁ HIGH |
| Change in functional  ability | Whitehead et al(2016) | 30 | 6m | Median (SE)  Not reported | Median (SE)  Not reported | Median difference (SE) (95% CI)  *NEADL scale: 0–66 (highest best)  1.58 (5.28) −9.47 to 12.64  *Barthel index scale 0-20(highest best) 0.28 (1.12) −2.06 to 2.61 | ⨁⨁⨁⨁ HIGH |
| Change in functional  ability | Hattori et al (2019) | 375 | 4m | n= (%)  ind=3/46 (6.5%)  dep=9/58 (15.5%)  dep in 2 or more=9/86 (10.5%) | n= (%)  Ind=1/44 (2.3%)  dep =1/60 (1.7%)  dep in 2 or more=5/81 (6.2%) | n=difference % (95%CI) *binary  Scale  ind=4.2 % (-4.1 to 12.6)  dep =13.9% (4.0 to 23.7)  dep in 2 or more= 4.3 % (-4.0 to 12.6) | ⨁⨁⨁◯ MODERATE |
| Change in quality of life | Tuntland et al  (2015) | 61 | 3m | mean (95%ci)  *Coop/Wonka  scale 1 to 5 (highest best) daily activities:  2.7 (2.3 to 3.1) | Mean (95%CI)  2.9 (2.5 to 3.2) | Mean difference (95%CI)  -0.4 (-0.9 to 0.2) p=0.21 | ⨁⨁⨁⨁ HIGH |
| Change in quality of life | Tuntland et al  (2015) | 61 | 6m | mean (95%ci)  *Coop/Wonka  scale 1 to 5 (highest best) daily activities:  2.5 (2.0-3.0) | mean (95%ci)  2.8 (2.3-3.2) | Mean difference (95%CI)  −0.4 (−0.3-0.5) p=0.22 | ⨁⨁⨁⨁ HIGH |
| Change in quality of life | Whitehead et al (2016) | 30 | 3m | Median (SE)  Not reported  /15 | Median (SE)  Not reported  /15 | Median difference (SE) (95% CI) **  EQ5D (scale: −0.11 to 1) −0.03 (0.15) −0.35 to 0.28  ASCOT (scale: 0–1): 0.06 (0.11) −0.18 to 0.30  SF-36 PCS (scale: 0–100): 1.52 (4.75) −8.43 to 11.47  SF-36 MCS (scale: 0–100): 7.84 (3.17)1.17 to 14.51*  *(1 outlier removed)  ** all scales highest best | ⨁⨁⨁⨁ HIGH |
| Change in quality of life | Whitehead et al (2016) | 30 | 6m | Median (SE)  Not reported  /15 | Median (SE)  Not reported  /15 | Median difference (SE) (95% CI) **  EQ5D (scale: −0.11 to 1) 0.23 (0.22) −0.23 to 0.69  ASCOT (scale: 0–1): 0.04 (0.10) −0.17 to 0.25  SF-36 PCS (scale: 0–100): 0.09 (5.33) −11.06 to 11.24  SF-36 MCS (scale: 0–100): 3.39 (4.90) −6.88 to 13.66  ** all scales highest best | ⨁⨁⨁⨁ HIGH |
| Change in functional mobility | King et al (2012) | 186 | 4m | Mean (SE)  22.3 (1.1)  *Timed up and go (in seconds) | Mean (SE)  22.3(2.0) | Mean difference (95%CI) and p value  Not reported | ⨁⨁⨁⨁ HIGH |
| Change in functional mobility | King et al (2012) | 186 | 7m | Mean (SE)  22.6(1.5)  *timed up and go (seconds) | Mean (SE)  22.4 (2.0) | Mean difference (SE) (95% CI) and p value -0.1(-4.2 to 4.1)  P=0.98 | ⨁⨁⨁⨁ HIGH |
| Change in functional mobility | Parsons et al  (2013) | 205 | 6m | Least means squares +or- SE (95%CI)  6.8 +- 0.44 (6.3 TO 7.69) | Least means squares +or- SE (95%CI)  6.14+-0.52 (5.60 to 7.33) | Least means squares +or- SE (95%CI) p value  P= 0.003 | ⨁⨁⨁◯ MODERATE |
| Change in functional mobility | Tuntland et al  (2015) | 61 | 3m | Mean (95%CI)  19.6 (14.2 to 25.1)  * timed up and go (in seconds) | Mean (95%CI)  17.9 (14.0 to 21.8) | Mean difference (95%CI) and p value  -0.4(-4.4 to 3.5)  P=0.82  Adjusted effect size=0.1 | ⨁⨁⨁⨁ HIGH |
| Change in functional mobility | Tuntland et al  (2015) | 61 | 9m | Mean (95%CI)  *19.9 (14.7 to 25.0)  * timed up and go (time in seconds) | Mean (95%CI)  18.1 (13.4 to 22.8) | Mean difference (95%CI)  0.3 (-3.7 to4.4)  adjusted effect size=  0.88 | ⨁⨁⨁⨁ HIGH |
| Adverse effect | Hattori et al (2019) | 375 | 3m | 20/190 (10.5%) | 16/185 (8.6%) | P=0.659 | ⨁⨁⨁◯ MODERATE |

*n=number, t=time * The cumulative evidence synthesis was coherently summarised to determine certainty of the evidence using the definitions established by GRADE (GradePro; 2020).

**References for supplementary appendix**

Barton GR, Sach, T. H., Doherty, M., Avery, A. J., Jenkinson, C., & Muir, K. R. (2007) An assessment of the discriminative ability of the EQ-5Dindex, SF-6D, and EQ VAS, using sociodemographic factors and clinical conditions. *The European Journal of Health Economics*. 9:237-49. doi: doi:10.1007/s10198-007-0068-z

Bergström A, Borell L, Meijer S, Guidetti, S. (2019) Evaluation of an intervention addressing a reablement programme for older, community-dwelling persons in Sweden (ASSIST 1.0): a protocol for a feasibility study. *BMJ Open*; 9(7).

Bohannon RW, Bear-Lehman J, Desrosiers J, Massy-Westropp N, Mathiowetz V.(2007) Average grip strength: a meta-analysis of data obtained with a Jamar dynamometer from individuals 75 years or more of age. *J Geriatr Phys Ther*. 30(1):28–30.

Cameron ID, Fairhall N, Langron C, et al.(2013) A multifactorial interdisciplinary intervention reduces frailty in older people: randomized trial. *BMC medicine*. 11:65. doi: <http://dx.doi.org/10.1186/1741-7015-11-65>. PubMed PMID: 1427001847; 23497404.

Carswell A, McColl MA, Baptiste S, Law M, Polatajko H, Pollock N. (2004) The Canadian Occupational Performance Measure: a research and clinical literature review. *Can J Occup Ther*. 71(4):210-22. doi: 10.1177/000841740407100406. PMID: 15586853.

Chiu T, & Oliver, R. (2006) Factor analysis and construct validity of the SAFER-HOME . *Occupational Therapy Journal of Research.* 6:132-42

Clare LA, Kudlicka JR, Oyebode RW, Jones A, Bayer I, Leroi M et al. (2009) Individual goal-oriented cognitive rehabilitation to improve everyday functioning for people with early-stage dementia: A multicentre randomised controlled trial (the GREAT trial). *International Journal of Geriatric Psychiatry*  34(5): 709-721.

Colin C., Wade D., Davies S. & Horne V. (1988) The Barthel ADL Index: a reliability study. *International Disability Studies* 10, 61–63

Fairhall N, Sherrington C, Lord S, Susan K, Cameron ID.(2015) A multifactorial interdisciplinary intervention reduces frailty, increases function and is cost-effective in older adults who are frail: randomised controlled trial. *Physiotherapy*. 101:e371-e2. doi: <https://doi.org/10.1016/j.physio.2015.03.588>

Gitlin LN, Hauck WW, Dennis MP, Winter L, Hodgson N, Schinfeld S.(2009) Long-Term Effect on Mortality of a Home Intervention that Reduces Functional Difficulties in Older Adults: Results from a Randomized Trial. Journal of the American Geriatrics Society. 57(3): 476-481.

Gitlin LN, Winter L, Dennis MP, Hodgson N, Hauck WW. (2010) A Biobehavioral Home-Based Intervention and the Well-being of Patients With Dementia and Their Caregivers: The COPE Randomized Trial. *JAMA* 304(9): 983-991.

Gitlin, LN, Winter MP, Dennis M, Corcoran S, Schinfeld S, Hauck W.(2006) A Randomized Trial of a Multicomponent Home Intervention to Reduce Functional Difficulties in Older Adults. *Journal of the American Geriatrics Society* 54(5); 809-816.

GradePro (2021) [www.gradepro.org](http://www.gradepro.org) available at <https://www.gradepro.org/> accessed on: 01.03.21

Guralnik JM, Simonsick EM, Ferrucci L, Glynn RJ, Berkman LF, Blazer DG. (1994) A short physical performance battery assessing lower extremity function: association with self-reported disability and prediction of mortality and nursing home admission*. J Gerontol* 49:M85-94.

Haley S, Jette A, Coster W, et al. (2002) Late Life Function and Disability Instrument: II. Development and evaluation of the function component. *J Gerontol Med Sci.* 57A:M217–M222.

Jeon Y-H, Simpson JM, Low L-F, Woods R, Norman R, Mowszowski L, et al.(2019) A pragmatic randomised controlled trial (RCT) and realist evaluation of the interdisciplinary home-bAsed Reablement program (I-HARP) for improving functional independence of community dwelling older people with dementia: an effectiveness-implementation hybrid design. *BMC geriatrics* 19(1):[199 p.] <https://doi.org/10.1186/s12877-019-1216-x>

Kempen GIJM, Yardley, L., van Haastregt, C. M., Ziljstra, R., Beyer, N., & Hauer, K. (2008) The Short FES-I: a shortened version of the falls efficacy scale-international to assess fear of falling. *Age and Ageing*; 37:45-50. doi: doi:10.1093/ageing/afm157.

Kessler D, Egan M, Dubouloz C-J, McEwen S, Graham FP.(2017) Occupational Performance Coaching for Stroke Survivors: A Pilot Randomized Controlled Trial. The American Journal of Occupational Therapy. 71(3):1-7. doi: <http://dx.doi.org/10.5014/ajot.2017.024216>. PubMed PMID: 1890809455.

Kinnersley P, Peters T, Stott N. (1995) Measuring functional health status in primary care using the COOP-WONCA charts: Acceptability, range of scores, construct validity, reliability and sensitivity to change. *The British journal of general practice*  44:545-9.

Koenig H.G., Westlund R.E., George L.K., Hughes D.C., Blazer D.G. & Hybels C. (1993) Abbreviating the Duke Social Support Index for use in chronically ill elderly individuals. *Psychosomatics.* 34:61-9.

Liu C-j, Xu H, Keith NR, Clark DO. (2017) Promoting ADL independence in vulnerable, community-dwelling older adults: a pilot RCT comparing 3-Step Workout for Life versus resistance exercise. *Clinical Interventions in Aging*. 12:1141-9. doi: <http://dx.doi.org/10.2147/CIA.S136678>. PubMed PMID: 2224588535.

Mackintosh S.(2009) Functional independence measure. *Aust J Physiother.* 55(1):65. doi: 10.1016/s0004-9514(09)70066-2. PMID: 19226247.

Metzelthin SF, Rooijackers TH, Zijlstra GAR, van Rossum E, Veenstra MY, Koster A, et al.(2018) Effects, costs and feasibility of the ‘Stay Active at Home’ Reablement training programme for home care professionals: study protocol of a cluster randomised controlled trial. *BMC Geriatrics*. 18(1):276. doi: 10.1186/s12877-018-0968-z.

Nouri FM, Lincoln NB.(1987) An extended activities of daily living scale for stroke patients. *Clinical Rehabilitation*. 1:301-5.

Page, S J, Dunning K, Hermann V, Leonard A, Levine P. (2011) Longer versus shorter mental practice sessions for affected upper extremity movement after stroke: a randomized controlled trial. *Clinical Rehabilitation* 25(7): 627-637.

Parsons M, Senior H, Kerse N, Chen M-h, Jacobs S, Anderson C. (2017) Randomised trial of restorative home care for frail older people in New Zealand. *Nursing Older People* (2014+).29(7):27. doi: <http://dx.doi.org/10.7748/nop.2017.e897>. PubMed PMID: 1938604162.

Podsiadlo D. & Richardson S. (1991) The timed ‘‘Up & Go’’: a test of basic functional mobility for frail elderly persons. *Journal of the American Geriatrics Society* 39, 142– 148.

PSSRU (2018) Adult Social Care Outcomes Toolkit. Personal Social Service Research Unit at University of Kent [www.pssru.ac.uk](file:///C:\Users\mszfma\AppData\Local\Microsoft\Windows\INetCache\Content.Outlook\K3ZIW1K8\www.pssru.ac.uk) (online). available at: <https://www.pssru.ac.uk/ascot/>

Schwartz JK, Grogan KA, Mutch MJ, Nowicki EB, Seidel EA, Woelfel SA, et al.(2017) Intervention to Improve Medication Management: Qualitative Outcomes From a Phase I Randomized Controlled Trial. *The American Journal of Occupational Therapy*. 1(6):1-10. doi: <http://dx.doi.org/10.5014/ajot.2017.021691>. PubMed PMID: 1964552840.

Senior HEJ, Parsons M, Kerse N, et al (2014) Promoting independence in frail older people: a randomised controlled trial of a restorative care service in New Zealand. *Age and Ageing.* 2014;43(3):418-24. doi: 10.1093/ageing/afu025.

Steffen TM, Hacker TA, Mollinger L (2002). Age-and gender-related test performance in community-dwelling elderly people: Six-Minute Walk Test, Berg Balance Scale, Timed Up & Go Test, and gait speeds. *Phys Ther*. 82(2):128–37.

Szanton SL, Wolff JW, Leff B, Thorpe RJ, Tanner EK, Boyd C, et al. (2014) CAPABLE trial: a randomized controlled trial of nurse, occupational therapist and handyman to reduce disability among older adults: rationale and design. *Contemporary clinical trials*. 38(1):102-12. Epub 2014/03/28. doi: 10.1016/j.cct.2014.03.005. PubMed PMID: 24685996.

Voigt-Radloff S, Graff M, Leonhart R, Schornstein K, Jessen F, Bohlken J, et al.(2011) A multicentre RCT on community occupational therapy in Alzheimer's disease: 10 sessions are not better than one consultation. *BMJ Open*. 1(1). doi: <http://dx.doi.org/10.1136/bmjopen-2011-000096>. PubMed PMID: 1783499233

Ware JE, Kosinski M, Dewey JE. (2000) How to Score Version Two of the SF-36 Health Survey. Lincoln, OR.

Yoshida I, Hirao K, Nonaka T. (2018) Adjusting Challenge–Skill Balance to Improve Quality of Life in Older Adults: A Randomized Controlled Trial. *The American Journal of Occupational Therapy.* 72(1):1-8. doi: <http://dx.doi.org/10.5014/ajot.2018.020982>. PubMed PMID: 1981701904
